# A Meta-Analysis of Task-Based fMRI Studies on Alcohol Use Disorder

**DOI:** 10.3390/brainsci15070665

**Published:** 2025-06-20

**Authors:** Maxime Roberge, Mélanie Boisvert, Stéphane Potvin

**Affiliations:** 1 Centre de Recherche de l’Institut Universitaire en Santé Mentale de Montréal, Montreal, QC H1N 3V2, Canada; maxime.roberge.3@umontreal.ca (M.R.); melanie.boisvert.1@umontreal.ca (M.B.); 2 Department of Psychiatry and Addiction, University of Montreal, Montreal, QC H3T 1J4, Canada

**Keywords:** alcohol use disorder, meta-analysis, fMRI, cognition

## Abstract

**Background**: Previous syntheses on the neural effects of alcohol have been restricted to tasks assessing craving, cognitive control, and reward processing. Despite extensive research, a comprehensive synthesis of functional magnetic resonance imaging (fMRI) findings on alcohol use disorder (AUD) remains lacking. This study aimed to identify consistent brain activation alterations across all cognitive and emotional tasks administered to individuals with AUD while distinguishing between short-term and long-term abstinence and using activation likelihood estimation meta-analysis. Sub-analyses on task types were performed. **Methods:** A systematic review identified 67 fMRI studies on participants with an AUD. **Results:** The meta-analysis revealed significant alterations in brain activity, including both hypo- and hyperactivation in the left putamen across all AUD participants. These alterations were observed more frequently during decision-making and reward tasks. Short-term abstinent individuals exhibited hypoactivation in the right middle frontal gyrus (MFG), corresponding to the dorsolateral prefrontal cortex. In contrast, long-term abstinent individuals displayed hypoactivation in the right superior frontal gyrus (SFG) and dorsal anterior cingulate cortex (dACC). This meta-analysis highlights critical neural alterations in AUD, particularly in regions associated with reward processing (putamen), executive functions (MFG and SFG), and attentional salience (dACC). Putamen changes were predominantly observed during short-term abstinence and in decision-making, as well as reward processing tasks. dACC and SFG hypoactivation were specific to long-term abstinence, while MFG hypoactivation was specific to short-term abstinence. **Conclusions:** These findings support prior research indicating a motivational imbalance and persistent executive dysfunctions in AUD. Standardizing consumption metrics and expanding task diversity in future research is essential to further refine our understanding of the neural effects of AUD.

## 1. Introduction

Alcohol is one of the most consumed psychoactive substances in the world [1]. Nearly 400 million people live with an alcohol-related disorder around the world, of which 209 million suffer from dependence, resulting in approximately 2.6 million annual deaths [2]. In the United States of America, the costs associated with alcohol, including accidents, lost productivity, and healthcare utilization, have reached USD 249 billion over the last decade [3,4,5]. Heavy and chronic alcohol use raises the probability of experiencing anxiety, depression, and bulimia nervosa and adopting violent or suicidal behaviors [6,7]. Finally, chronic alcohol use increases the risk of neurodegenerative diseases, such as Alzheimer’s disease [8]. Due to the addiction liability of alcohol and its psychiatric and cognitive effects, several neuroimaging studies have been pursued to understand the effects of chronic alcohol use on the brain.

Structural neuroimaging research has demonstrated that chronic alcohol use is associated with brain tissue shrinkage, evidenced by reduced cortical thickness, particularly in the frontal and temporal lobes, as well as in the cingulate gyrus [1,9,10]. Voxel-based morphometry studies have revealed that key regions of the mesolimbic system, namely, the putamen and the nucleus accumbens, are particularly impaired in chronic alcohol users [9,11,12]. Diffusion neuroimaging has shown that chronic alcohol use is associated with alterations to the integrity of white matter fibers, notably in the corpus callosum, cingulum, and fornix [13]. On the other hand, the involvement of corticospinal and thalamic tracts remains debated [14]. Chronic alcohol use impairs gray matter in (meso)-limbic and frontotemporal circuits, involved in reward and memory, and white matter fibers, known to be involved in cognition, emotion, and self-processing [10]. While some of these alterations may be mitigated by abstinence, the variability of results across studies complicates conclusions about the persistency of the observed changes [14,15].

As for functional neuroimaging, most task-based research using functional magnetic resonance imaging (fMRI) has focused on experimental paradigms assessing craving (with alcohol-related cues) [16,17,18,19], executive functions [17,18,20], and reward processing [17,21]. While meta-analyses on cravings have revealed that alcohol is associated with dysfunctions in the brain reward system, the default-mode network, and parieto-occipital regions, meta-analyses on executive functions (e.g., cognitive control) have revealed alterations in frontal and cingulate regions, and meta-analyses on reward processing have revealed expected alterations in brain reward regions [17,18,19,20,21]. Echoing the results of the task-based fMRI literature, research syntheses on the functional connectivity literature have shown that alcohol is associated with impaired functional connectivity between the ventral striatum and the ventromedial prefrontal cortex (vmPFC), which are core regions of the brain reward system [22]. Despite the strengths of these meta-analytic findings, results have been heterogeneous and sometimes inconsistent across meta-analyses. The heterogeneity of findings may be explained by the heterogeneity of the included populations. While some syntheses have included only studies on alcohol use disorder (AUD) [20], others have also included studies on binge or heavy drinking [16,18,19,21]. In addition, in most previous syntheses, studies with no group of healthy controls have been included [16,17,18], making it difficult to determine if the observed patterns of activations are abnormal or not. Another issue of previous syntheses is that most of them have included studies using predefined regions of interest (ROIs) [16,17,19,20,21], although this is not recommended in the fMRI meta-analytic guidelines [23], as this approach may skew results toward a priori hypotheses. Furthermore, some meta-analyses have included studies on substances other than alcohol [16,17], making it difficult to determine if the results are related to alcohol per se or not. A final limitation of previous syntheses is that sub-analyses on abstinence have been performed in only a minority of cases [19], leaving the question of the potential reversibility of alcohol effects unanswered.

In addition to tasks assessing cravings, executive functions, and reward processing, several fMRI studies have examined alternative experimental paradigms, including tasks assessing negative emotion processing, decision-making, language, memory, auditory functions, and social–cognitive processes [24,25,26,27,28]. This interest stems from the high co-occurrence between chronic alcohol use and anxio-depressive symptoms [29], as well as the fact that AUD is associated with cognitive deficits that are not restricted to executive functions. Indeed, two large meta-analyses have shown that AUD is associated with deficits in attention, decision-making, psychomotor speed, social cognition, and verbal fluency (language) [30,31]. Unfortunately, previous meta-analyses of task-based fMRI on chronic alcohol use have not included studies examining emotion processing, decision-making, language, memory, auditory functions, or social cognitive processes in the scanner. This has prevented a comprehensive synthesis of the neural alterations observed in chronic alcohol users in the entirety of the task-based fMRI literature. In theory, the addition of the full range of tasks that have been used in the field may help unravel widespread neural alterations, extending the alterations that have been observed in brain reward and executive regions in previous syntheses [17,18,19,20,21]. This assumption is consistent with the fact that alcohol produces its psychoactive effects primarily via glutamatergic and GABAergic receptors that are found in high concentrations across the brain [1].

In view of the state of knowledge, we propose to perform a meta-analysis of task-based fMRI studies on alcohol, regardless of the task used in the scanner. This across-task approach has already been successfully applied in the context of several mental disorders, including borderline personality disorder, major depressive disorder, bipolar disorder, posttraumatic stress disorder, anxiety disorders, and neurodevelopmental disorders [32,33,34,35,36,37]. Secondary analyses of task types will be performed. To reduce the heterogeneity of the population, we will include studies involving participants with AUD, compared to a control group of healthy volunteers, or studies adopting dimensional analyses of AUD severity levels (from recreational use to disorder). Also, we will only include studies having used a whole-brain analysis approach, excluding predefined ROIs, to provide the most comprehensive overview of the neural effects of alcohol. By doing so, we expect to observe neural alterations that are more widespread than the alterations reported in previous fMRI syntheses on alcohol.

## 2. Methods

### 2.1. Literature Search and Selection Criteria

An exhaustive search of PubMed, Web of Science, Google Scholar, and Embase was performed independently by two authors (MR and MB) with the following keywords: ‘Alcohol Use Disorder’ and ‘AUD’ and ‘fMRI’ and ‘Task’. In addition, cross-referencing of the most recent and large-scale meta-analyses in the field was conducted [16,17,18,19] to ensure the inclusion of all relevant studies.

The selected articles were then reviewed by all authors (SP, MB, and MR) to affirm the validity of the studies in the present research. To ensure the quality of the selected articles, rigorous exclusion criteria were chosen. These criteria included binge drinking; heavy drinking; or any other type of alcohol consumption that was not supported by a DSM or AUDIT diagnosis, acute administration, or any other mental health or substance use disorder that was primary. Articles in a language other than English (or French) or which were incomplete were also excluded from our analysis. All these criteria were motives for exclusion. For studies originating from the same cohort (e.g., the Boystown program), we only included studies reporting results from distinct experimental tasks.

### 2.2. Data Extraction

Data extraction was performed by one author (MR) and was verified by the other authors (MB and SP). Data from the selected articles were extracted manually and included sociodemographic variables (the number of participants, the substance consumed and the age of each group, the duration of abstinence, and the diagnostic criterion), as well as the MRI analysis variables (power in tesla, smoothing [mm], voxel size [mm], repetition time [TR, ms], task, contrast used, direction of activation [hyper/hypo], the coordinates [x, y, z], and the stereotaxic atlas used [mainly MNI and TAL]).

### 2.3. Activation Likelihood Estimation

The activation likelihood estimation (ALE) method was employed for the coordinate-based meta-analysis using GingerALE version 3.0.2 (https://www.brainmap.org/ale/, accessed on 12 February 2024). This technique assesses spatial convergence across studies and requires that peak foci be provided in stereotactic coordinates [38]. Consequently, coordinates in the ‘x, y, z’ format were extracted from each article. These coordinates were then converted from Talairach to Montreal Neurological Institute (MNI) coordinates using the icbm2tal transform [39].

The main meta-analysis was conducted by combining long-term abstinent (more than 4 weeks abstinent) and short-term abstinent participants with AUD (less than four weeks abstinent). As in previous studies on the neurocognitive effects of alcohol, 4 weeks (28 days) seems to be the best variable to discriminate notable differences in abstinence [40]. Sub-analyses were also conducted for the short-term and long-term abstinence studies. The ALE approach does not account for the direction of effects. Therefore, for each group of experiments (short- and long-term abstinence, total), three main separate analyses were conducted, one for the hyperactivated group of foci, one for the hypoactivated group of foci, and one combining both the hypoactivated and hyperactivated foci groups, as in several past meta-analyses using ALE [16,17,21]. For each analysis, activation maps were modeled for each foci group using a mask, the foci, and a Gaussian blur. The full width at half-maximum (FWHM) of the Gaussian filter was determined based on the sample size of each study, incorporating spatial uncertainty associated with each foci group [41]. An unthresholded ALE image was generated from the union of the modeled activation maps. ALE scores were then compared against a null distribution using a histogram, and a table of *p*-values was produced. This unthresholded ALE image and *p*-value table were used to create a 3D *p*-value image, which was then thresholded for statistical significance using family-wise error (FWE) correction [41]. The uncorrected *p*-value threshold was set at *p* < 0.001, with cluster-level FWE correction applied at *p* < 0.05 using 1000 permutations. Significant results were functionally characterized using NeuroSynth meta-analytic term-based decoding, and the 5 most strongly correlated terms were identified.

Since meta-regression analyses are not supported by GingerALE, alternative steps were taken to examine the moderating effects of age, sex ratio, MRI magnetic field strength (1.5T vs. 3T), MRI parameters (voxel size, time repetition, smoothing level, and number of tesla), and task types [craving, executive functions (e.g., attention, cognitive control, and working memory), emotion processing, decision-making, reward processing, and other functions assessed in fewer than 5 studies (language, audition, etc.)] on the probabilities of activation of each significant cluster. To accomplish this, probabilities of activation for each study were extracted and analyzed using SPSS Version 28. Binary logistic regressions were conducted for each confounding variable to assess its impact on the probabilities of activation (0 or 1) of significant clusters. These analyses were performed individually for each predictor and each significant cluster.

## 3. Results

### 3.1. Included Studies

Out of 3090 studies identified (after duplicates were removed), 2883 were excluded based on their abstracts. Of the remaining 257 articles, 190 were further excluded for the following reasons: (1) the analyses focused on predetermined regions of interest, (2) incomplete data, (3) a lack of between-group comparisons or results, (4) duplicate cohorts, (5) a reliance on connectivity results, (6) the absence of a control group, (7) the use of acute administration, (8) conference summaries, (9) involvement of binge/heavy drinkers, or (10) alcohol not being the primary diagnosis (Supplementary Figure S1 and Table S1). A total of 67 studies with a sample size of 2421 subjects met the final inclusion criteria (Table 1). Studies used tasks assessing craving, reward processing, executive functions, emotion processing, decision-making, language, memory, auditory functions, and social cognitive processes (Supplementary Table S2). The 67 studies were subdivided into studies involving short-term and long-term abstinent samples, apart from 4 studies that could not categorized considering that they involved mixed samples, with some patients with short-term and others with long-term abstinence.

#### 3.1.1. Hypoactivation and Hyperactivation Pooled Together in the Whole Sample (Short-Term and Long-Term Abstinence)

This analysis included 730 foci from 67 experiments on 2421 subjects with an alcohol use disorder. Aberrant activity of the left putamen (68.1%), caudate body (22.5%), and caudate head (9.3%) was found to be significant at a corrected threshold of *p* < 0.05 (Table 2). Associations with terms like delay, gains, monetary, incentive, and losses were found in Neurosynth.

#### 3.1.2. Hypoactivated Foci Group in the Whole Sample

Analysis of the hypoactivated foci group included 345 foci from 44 experiments on 1245 subjects with an alcohol use disorder. No clusters of significant decrease activation were found at a corrected threshold.

#### 3.1.3. Hyperactivated Foci Group in the Whole Sample

For the hyperactivated foci group analysis, 385 foci were included, comprising 47 experiments on 1759 subjects with an alcohol use disorder. No clusters of significant increase activation were found at a corrected threshold.

#### 3.1.4. Hypoactivation and Hyperactivation Pooled Together in the Short-Term Abstinent Sample

**Figure 1:**
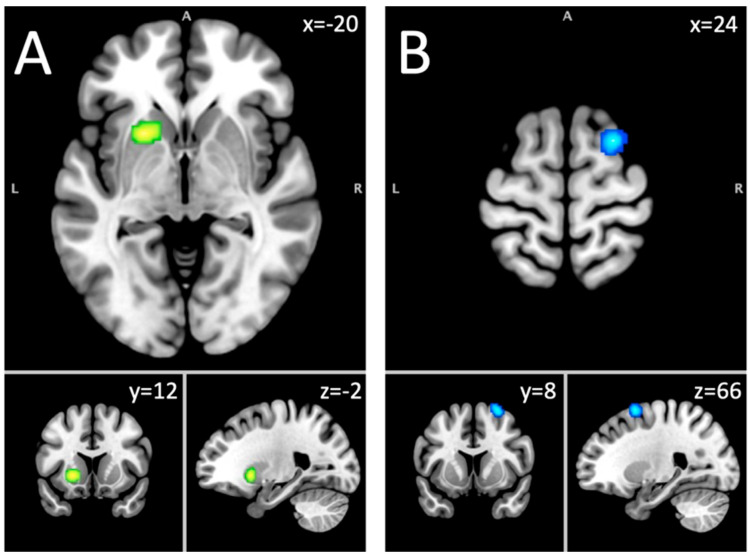
Short-term abstinence fMRI results. Note. (**A**) = putamen (hyper- and hypoactivation); (**B**) = middle frontal gyrus (hypoactivation).

This analysis included 349 foci from 37 experiments on 1097 subjects with an alcohol use disorder. Aberrant activity of the left putamen was found to be significant at a corrected threshold of *p* < 0.05 (Table 2; Figure 1). Since this cluster was the same as the one found in the hyper–hypoactivated foci analysis in the combined sample, the Neurosynth terms were the same, as mentioned previously.

#### 3.1.5. Hypoactivated Foci Group in the Short-Term Abstinent Sample

The analysis of hypoactivated foci included 125 foci from 25 experiments on 631 subjects with an AUD. Decreased activation of a cluster located in the right middle frontal gyrus (MFG) (56.7%), the superior frontal gyrus (SFG) (40%), and the sub-gyral (3.3%) was found in participants with alcohol use disorder compared to controls (Table 2; Figure 1). This cluster was associated with Neurosynth terms like response times, task difficulty, instructions, difficulty, and signal tasks.

#### 3.1.6. Hyperactivated Foci Group in the Short-Term Abstinent Sample

For the hyperactivated foci group analysis, 224 foci were included, comprising 29 experiments on 893 subjects with an AUD. No clusters of significant increase activation were found at a corrected threshold.

#### 3.1.7. Hypoactivation and Hyperactivation Pooled Together in the Long-Term Abstinent Sample

Included are 348 foci from 26 experiments on 1001 subjects with an AUD. This analysis yielded no clusters.

#### 3.1.8. Hypoactivated Foci Group in the Long-Term Abstinent Sample

**Figure 2:**
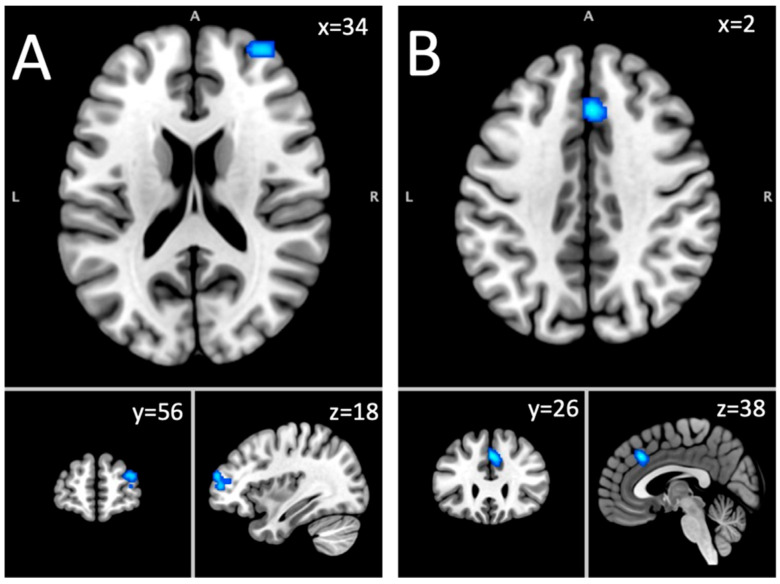
Long-term abstinence fMRI results. Note. (**A**) = superior frontal gyrus (hypoactivation); (**B**) = dorsal anterior cingulate cortex (hypoactivation).

Analysis of the hypoactivated foci group included 212 foci from 17 experiments on 532 subjects with an AUD. This analysis yielded two significant clusters: (1) a cluster located in the right SFG (63.6%) and the MFG (36.4%); (2) a bilateral cluster in the cingulate gyrus (87.1%) and the medial frontal gyrus (12.9%) (Table 2; Figure 2). The first cluster was linked to Neurosynth terms like noxious, executive, working, working memory, and abilities. The second cluster was associated with terms like monitoring, task, error, learning task, and painful.

#### 3.1.9. Hyperactivated Foci Group in the Long-Term Abstinent Sample

For the hyperactivated foci analysis, 136 foci were included, comprising 16 experiments on 615 subjects with an AUD. No clusters of significant increase activation were found at a corrected threshold.

### 3.2. Sub-Analyses on Sociodemographic Variables, MRI Parameters, and Task Types

No associations were found between sociodemographic variables, MRI parameters, and probabilities of activation in each significant cluster (Table 3). Relationships with task types were identified (Table 3). In the short-term sample, significant positive associations were observed between the putamen and decision-making tasks (OR = 9.33, 95% CI [1.27–68.60], *p* = 0.028), as well as reward tasks (OR = 30.00, 95% CI [2.33–386.33], *p* < 0.009). No significant relationships were found between the MFG and any task type. In the long-term abstinent sample, a significant positive association was identified between the SFG and reward tasks (OR = 9.00, 95% CI [1.03–78.57], *p* < 0.047). However, no significant associations were observed in the case of the cingulate gyrus.

## 4. Discussion

The objective of this meta-analysis was to provide a comprehensive overview of the neural effects of AUD through whole-brain fMRI analyses, regardless of the task used in the scanner, while distinguishing effects based on the duration of abstinence. The activity of the left putamen emerged as being disrupted in the analysis combining both hypo- and hyperactivations. A positive association was found between the aberrant activity of the putamen and decision-making and reward tasks. In short-term abstinent AUD participants, hypoactivation was observed in the right MFG, corresponding to the dorsolateral prefrontal cortex (dlPFC). For long-term abstinent AUD participants, hypoactivation was observed in the right SFG encompassing the MFG, in the lateral part of the prefrontal cortex (PFC), and in the dorsal anterior cingulate cortex (dACC). In the whole sample of both short- and long-term AUD participants, the putamen was also found to be altered. Lastly, sub-analyses revealed no association between neural alterations and sociodemographic and MRI parameters.

Our results demonstrated that the activation of the left putamen is bidirectionally altered in AUD and that these alterations are mostly driven by short-term abstinence studies. As such, this result is consistent with previous structural neuroimaging studies that have reliably shown that gray matter volumes/concentrations are decreased in AUD patients [9,12,13,42]. Our findings are also consistent with previous fMRI meta-analyses, which have shown a similar pattern of complex alterations in the putamen in AUD [17,21]. In healthy volunteers, fMRI studies have demonstrated that this dopamine-rich structure is involved in reward processing and associative learning [43,44]. In AUD, previous fMRI reward studies have determined that the putamen shows decreased activation during the Monetary Incentive Delay Task [21] and hyperactivation in response to alcohol cues [17,18]. In the sub-analyses of task types, we observed associations between the putamen and reward processing and reward-based decision-making, which are consistent with the previous literature in the field. Taken together, the results from the current meta-analysis and previous meta-analyses strongly suggest that AUD is associated with a motivational imbalance, whereby the brain reward system becomes hyperactivated in response to alcohol cues while displaying a blunted response to rewarding stimuli or cues that are not alcohol-related (example: money) [45,46,47]. The fact that significant putamen alterations were only observed during short-term abstinence is consistent with the clinical literature having shown that dysphoric symptoms and alcohol cravings tend to stabilize during the first 3–4 weeks of alcohol withdrawal [48,49].

Two clusters in the right MFG and SFG were found to be hypo-activated in AUD, corresponding to the right dlPFC and the right lateral PFC. These results are consistent with past structural neuroimaging studies, which have demonstrated a reduction in gray matter in the MFG and SFG in AUD, with the dlPFC appearing to be predominantly affected [11,12]. In the healthy population, large-scale fMRI evidence has shown that the right MFG and SFG play critical roles in core executive functions, including response inhibition and working memory [50,51,52]. In agreement with these findings, we performed functional decoding analyses, which revealed that the right dlPFC cluster is associated with functions like response inhibition, whereas the right lateral PFC cluster is associated with functions like working memory. Some authors have argued that executive dysfunctions are central to the development and maintenance of AUD [53,54] since these deficits weaken the ability to plan and execute effective strategies, resulting in social and occupational problems, as well as impaired impulse regulation and a loss of behavioral control [55]. Supporting the proposed interaction between executive and reward regions in AUD, the sub-analyses on task types showed an association between the SFG and reward tasks. As synthesized by Stavro and colleagues (2013) and Crowe and colleagues (2019) [30,31], a large body of cognitive studies has shown that working memory and response inhibition are significantly impaired in AUD and that these deficits clearly persist after the first weeks of alcohol withdrawal, with residual effects sometimes lasting several months. The fact that the lateral PFC was found to be impaired in long-term abstinence studies is consistent with the cognitive literature on AUD.

Hypoactivation was observed in long-term abstinence studies in AUD in the dACC, which is one of the core hubs of the (attentional) salience network [56]. The alteration of the dACC is supported by the structural neuroimaging literature, which has consistently shown gray matter reductions in this region in substance use disorders, including AUD [11,12,13,57]. Our result is also consistent with the results of previous fMRI meta-analyses, which have consistently shown alterations in ACC activation in AUD in separate analyses of reward processing, craving, and executive functions [16,17,18,19,20,21]. In the healthy population, the dACC has been shown to be involved in top-down attention control and the detection of salient changes in the interoceptive and external environment [58,59]. In view of the well-known roles of the dACC, the reduced activity observed in this region in AUD patients may signal attentional biases toward alcohol-related stimuli and/or an inability to re-allocate attentional resources to non-alcohol stimuli [60]. The fact that dACC hypo-activation was only observed in long-term abstinence studies suggests that the dACC may play a key role in alcohol relapse, as recently proposed by some investigators [61].

This meta-analysis has significant strengths compared to previous fMRI meta-analyses in the field. We conducted a meta-analysis including only whole-brain fMRI studies involving participants with AUD specifically, as compared to healthy control participants. This approach allowed us to include a more homogeneous study population and to produce a more comprehensive overview of alcohol’s effects on the AUD population while reducing biases associated with the selection of regions of interest. Moreover, our meta-analysis is the first to quantitatively review all tasks available, including task domains that were excluded from previous syntheses (audition, language, social cognition, etc.). This approach allowed us to include 27 fMRI studies that would have been excluded had we limited the analyses to tasks assessing executive functions, reward processing, and craving (as in previous meta-analyses). Despite the variety of the tasks reviewed, our findings converge on alterations in brain regions involved in reward processing, executive functions, and attentional salience, suggesting that the neural alterations observed in AUD may be restricted to these psychological domains.

Despite these strengths, a few limitations need to be acknowledged. Although the selection of tasks was comprehensive, the number of studies per task domain was rather small in several cases, namely in the case of tasks assessing language, audition, motor functions, social cognition, and episodic memory. For these domains, the number of studies was lower than the minimal number of studies that has been estimated to provide sufficient statistical power for fMRI meta-analyses (e.g., 17 studies) [62,63]. Therefore, statistical power was most probably insufficient for all these domains. As such, this could explain why we failed to observe neural impairments in the brain regions/networks involved in these functions. Likewise, the lack of effect observed in nearly all sub-analyses must be taken cautiously, considering that they may be explained by a lack of power. For instance, we observed no differences between studies using 1.5T vs. 3T scanners, potentially suggesting that future studies may interchangeably resort to 1.5T scanners. It must be noted, however, that only 15 studies in the current meta-analysis used 1.5T scanners. Moreover, although sub-analyses were performed on several variables that may have influenced results (age, sex, MRI parameters, etc.), we were unable to examine the influence on the results of the quantities of alcohol consumed by AUD patients. Indeed, the included studies often lacked clear data on consumption in terms of standardized units and precise temporality, and some relied on subjective measures influenced by national and political standards. This is unfortunate considering that alcohol is presumed to be more neurotoxic for those who have consumed larger quantities in their lifetime, although results on this topic are more complex than previously thought [64,65,66].

The current study sought to identify the neural alterations associated with AUD by performing a meta-analysis of whole-brain fMRI studies including all tasks available. We found that AUD was associated with neural impairments in brain regions involved in reward processing (the putamen), executive functions (the MFG), and attention salience (dACC). The length of abstinence influenced results, with striatal changes being driven by short-term abstinence studies and dACC changes being observed only in long-term abstinence studies. These findings underscore the importance of broadening the array of task domains in future fMRI research on AUD. Diversifying these domains may help move beyond the central triad of networks identified and more thoroughly examine the potential diffuse effects of AUD on brain functioning. Finally, it will be crucial to standardize, in future fMRI research, alcohol consumption units to facilitate data conversion and comparison and, thus, refine our understanding of the neural effects of AUD.

## Figures and Tables

**Table 1:** Demographics.

n Cases	n Controls	Mean Age Cases	Mean Age Controls	% of Males for Cases	% of Males for Controls	Mean Days of Abstinence (Range)
Whole sample						
2421	1458	36.94	37.07	76.73	73.26	189.54 (5–2994) ^a^
Short-term abstinent sample						
1097	653	35.71	34.46	76.32	70.71	16.10 (5–25.30) ^b^
Long-term abstinent sample						
991	488	37.42	40.15	77.56	78.35	416.47 (34–2994) ^c^

Note. ^a^ = information available only for 44/67 studies; ^b^ = information available only for 23/37 studies; ^c^ = information available only for 19/26 studies.

**Table 2:** Results of the ALE meta-analysis.

Regions	L/R	Cluster Size (mm^3^)	ALE Value	Z-Score	Coordinates (MNI)
Hyper–hypoactivation combined: whole sample					
Putamen, caudate body, caudate head	L	1840	0.0367	5.66	−20, 12, −2
Hyperactivation: whole sample					
No clusters found					
Hypoactivation: whole sample					
No clusters found					
Hyper–hypoactivation combined: short-term abstinent sample					
Putamen	L	1096	0.0236	4.88	−20, 12, −2
Hyperactivation: short-term abstinent sample					
No clusters found					
Hypoactivation: short-term abstinent sample					
Middle frontal gyrus, superior frontal gyrus, sub-gyral	R	856	0.016	4.48	24, 8, 66
Hyper–hypoactivation combined: long-term abstinent sample					
No clusters found					
Hyperactivation: long-term abstinent sample					
No clusters found					
Hypoactivation: long-term abstinent sample					
Superior frontal gyrus, middle frontal gyrus	R	856	0.0147	3.85	34, 56, 18
Cingulate gyrus, medial frontal gyrus	R & L	800	0.017	4.26	2, 26, 38

Note. L = left; R = right; MNI = Montreal Neurologic Institute.

**Table 3:** Binary logistic regressions for confounding variables and probabilities of activation of each cluster.

		95 C.I. for Odds Ratio		
Predictors	Odds Ratio	Lower	Higher	*p*-Value
Hyper–hypoactivation in the short-term abstinent sample: putamen				
Age	0.985	0.900	1.077	0.735
Sex ratio (% male)	0.981	0.951	1.013	0.245
Days of abstinence	0.939	0.746	1.182	0.590
MRI field strength	0.737	0.110	4.955	0.753
Smoothing level	0.701	0.422	1.164	0.170
Voxel size	1.047	0.993	1.103	0.088
Time repetition	0.998	0.996	1.000	0.115
Craving studies	0.489	0.050	4.793	0.539
Decision-making studies	9.333	1.270	68.597	0.028 *
Emotion studies	1.350	0.124	14.734	0.806
Executive function studies	0.364	0.038	3.518	0.382
Reward processing studies	30.000	2.330	386.325	0.009 *
Other task studies	3.375	0.459	24.837	0.232
Hypoactivation in the short-term abstinent sample: middle frontal gyrus				
Age	0.942	0.854	1.040	0.234
Sex ratio (% male)	0.985	0.951	1.021	0.417
Days of abstinence	0.859	0.664	1.113	0.251
MRI field strength	0.762	0.060	9.611	0.833
Smoothing level	1.068	0.648	1.760	0.797
Voxel size	1.054	0.989	1.123	0.108
Time repetition	1.000	0.999	1.001	0.924
Craving studies	0.000	0.000	0.000	0.999
Decision-making studies	7.250	0.786	66.842	0.080
Emotion studies	0.000	0.000	0.000	0.999
Executive function studies	2.300	0.283	18.705	0.436
Reward processing studies	3.333	0.259	42.925	0.356
Other task studies	1.867	0.160	21.742	0.618
Hypoactivation in the long-term abstinent sample: superior frontal gyrus				
Age	0.963	0.894	1.038	0.323
Sex ratio (% male)	0.993	0.950	1.037	0.746
Days of abstinence	1.002	0.999	1.004	0.153
MRI field strength	0.500	0.066	3.770	0.501
Smoothing level	0.586	0.278	1.233	0.159
Voxel size	0.980	0.939	1.022	0.337
Time repetition	1.001	0.999	1.004	0.267
Craving studies	0.000	0.000	0.000	0.999
Decision-making studies	0.000	0.000	0.000	1.000
Emotion studies	3.800	0.201	72.000	0.374
Executive functions studies	1.167	0.166	8.186	0.877
Reward processing studies	9.000	1.031	78.574	0.047 *
Other task studies	1.500	0.208	10.823	0.688
Hypoactivation in the long-term abstinent sample: cingulate gyrus				
Age	1.025	0.931	1.128	0.617
Sex ratio (% male)	1.022	0.964	1.084	0.456
Days of abstinence	0.997	0.990	1.004	0.387
MRI field strength	1.125	0.097	13.036	0.925
Smoothing level	0.956	0.450	2.031	0.906
Voxel size	0.999	0.948	1.052	0.955
Time repetition	1.000	0.997	1.002	0.708
Craving studies	1.500	0.122	18.441	0.751
Decision-making studies	0.000	0.000	0.000	1.000
Emotion studies	0.000	0.000	0.000	0.999
Executive functions studies	0.000	0.000	0.000	0.999
Reward processing studies	6.333	0.630	63.639	0.117
Other task studies	3.400	0.377	30.655	0.275

Note. MRI = magnetic resonance imaging. *, significant result (*p* < 0.05).

## Data Availability

All data used are available in the manuscript. No new data were generated.

## Supplementary Materials

The following supporting information can be downloaded at https://www.mdpi.com/article/10.3390/brainsci15070665/s1: Figure S1: PRISMA flowchart; Table S1: PRISMA 2020 Checklist; Table S2: Characteristics of the included studies.
