# Supplementary material for: A Meta-Analysis of Task-Based fMRI Studies on Alcohol Use Disorder
Source: Brain Sci. 2025 Jun 20;15(7):665. doi: 10.3390/brainsci15070665 (PMC12293344; doi:10.3390/brainsci15070665)
Supplement: Supplementary file 1 [file brainsci-15-00665-s001.zip › brainsci-3669559-supplementary.pdf]

Supplementary Figure 1. PRISMA flowchart

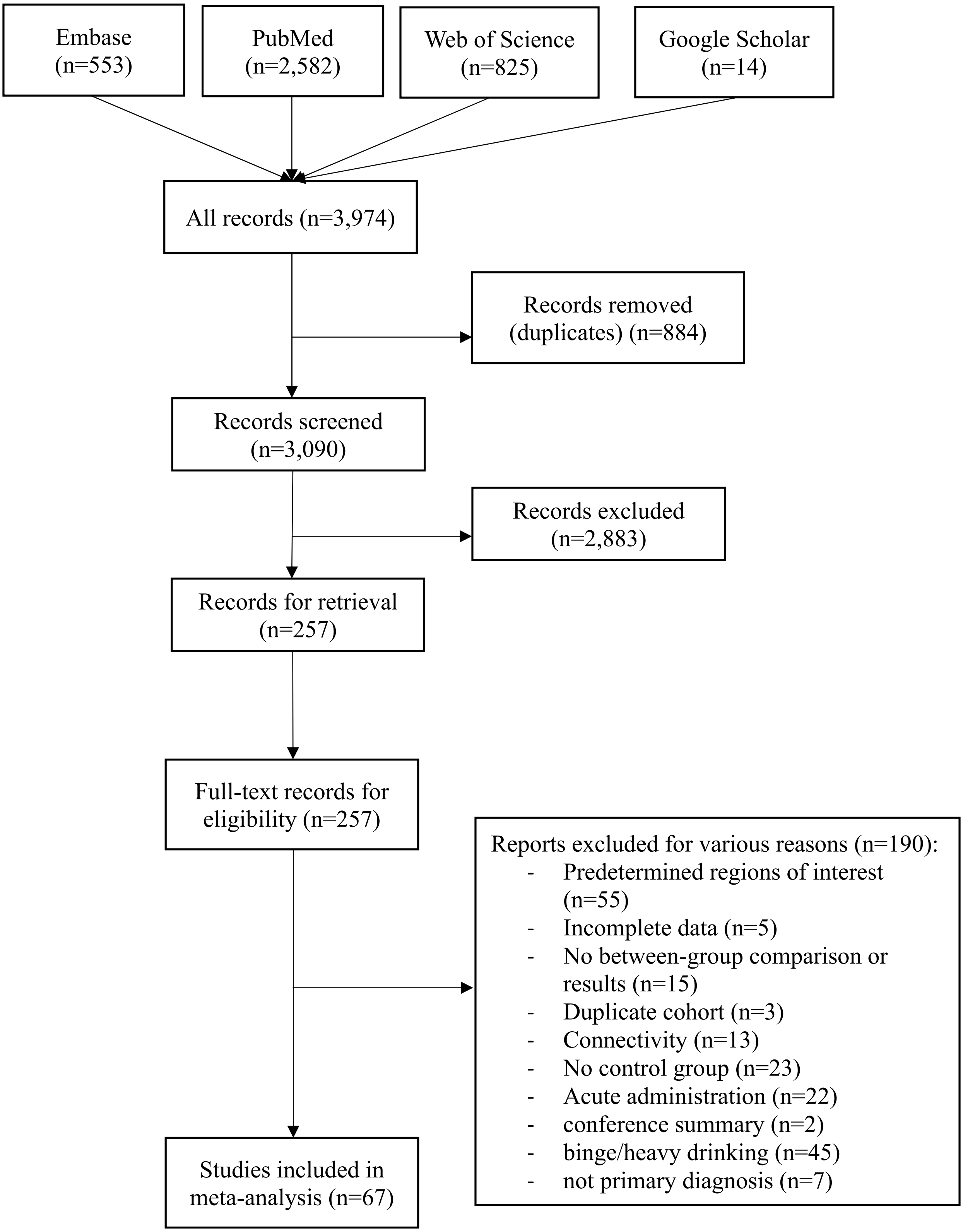

SUPPLEMENTARY TABLE 1: PRISMA 2020 CHECKLIST			
**Section and Topic**	**Item #**	**Checklist item**	**Location where item is reported**
**TITLE**			
Title	1	Identify the report as a systematic review.	1
**ABSTRACT**			
Abstract	2	See the PRISMA 2020 for Abstracts checklist.	2
**INTRODUCTION**			
Rationale	3	Describe the rationale for the review in the context of existing knowledge.	3-6
Objectives	4	Provide an explicit statement of the objective(s) or question(s) the review addresses.	6
**METHODS**			
Eligibility criteria	5	Specify the inclusion and exclusion criteria for the review and how studies were grouped for the syntheses.	7
Information sources	6	Specify all databases, registers, websites, organisations, reference lists and other sources searched or consulted to identify studies. Specify the date when each source was last searched or consulted.	7
Search strategy	7	Present the full search strategies for all databases, registers and websites, including any filters and limits used.	7
Selection process	8	Specify the methods used to decide whether a study met the inclusion criteria of the review, including how many reviewers screened each record and each report retrieved, whether they worked independently, and if applicable, details of automation tools used in the process.	7
Data collection process	9	Specify the methods used to collect data from reports, including how many reviewers collected data from each report, whether they worked independently, any processes for obtaining or confirming data from study investigators, and if applicable, details of automation tools used in the process.	7-8
Data items	10a	List and define all outcomes for which data were sought. Specify whether all results that were compatible with each outcome domain in each study were sought (e.g. for all measures, time points, analyses), and if not, the methods used to decide which results to collect.	7-8
	10b	List and define all other variables for which data were sought (e.g. participant and intervention characteristics, funding sources). Describe any assumptions made about any missing or unclear information.	7-8
Study risk of bias assessment	11	Specify the methods used to assess risk of bias in the included studies, including details of the tool(s) used, how many reviewers assessed each study and whether they worked independently, and if applicable, details of automation tools used in the process.	7
Effect measures	12	Specify for each outcome the effect measure(s) (e.g. risk ratio, mean difference) used in the synthesis or presentation of results.	7-8
Synthesis methods	13a	Describe the processes used to decide which studies were eligible for each synthesis (e.g. tabulating the study intervention characteristics and comparing against the planned groups for each synthesis (item #5)).	7-8
	13b	Describe any methods required to prepare the data for presentation or synthesis, such as handling of missing summary statistics, or data conversions.	7-8
	13c	Describe any methods used to tabulate or visually display results of individual studies and syntheses.	NA
	13d	Describe any methods used to synthesize results and provide a rationale for the choice(s). If meta-analysis was performed, describe the model(s), method(s) to identify the presence and extent of statistical heterogeneity, and software package(s) used.	8-9
	13e	Describe any methods used to explore possible causes of heterogeneity among study results (e.g. subgroup analysis, meta-regression).	8-9
	13f	Describe any sensitivity analyses conducted to assess robustness of the synthesized results.	8-9
Reporting bias assessment	14	Describe any methods used to assess risk of bias due to missing results in a synthesis (arising from reporting biases).	NA
Certainty assessment	15	Describe any methods used to assess certainty (or confidence) in the body of evidence for an outcome.	NA
**RESULTS**			
Study selection	16a	Describe the results of the search and selection process, from the number of records identified in the search to the number of	10

SUPPLEMENTARY TABLE 1: PRISMA 2020 CHECKLIST			
**Section and Topic**	**Item #**	**Checklist item**	**Location where item is reported**
		studies included in the review, ideally using a flow diagram.	
	16b	Cite studies that might appear to meet the inclusion criteria, but which were excluded, and explain why they were excluded.	NA
Study characteristics	17	Cite each included study and present its characteristics.	Suppl mat
Risk of bias in studies	18	Present assessments of risk of bias for each included study.	Suppl mat
Results of individual studies	19	For all outcomes, present, for each study: (a) summary statistics for each group (where appropriate) and (b) an effect estimate and its precision (e.g. confidence/credible interval), ideally using structured tables or plots.	Suppl mat
Results of syntheses	20a	For each synthesis, briefly summarise the characteristics and risk of bias among contributing studies.	10-13
	20b	Present results of all statistical syntheses conducted. If meta-analysis was done, present for each the summary estimate and its precision (e.g. confidence/credible interval) and measures of statistical heterogeneity. If comparing groups, describe the direction of the effect.	10-13
	20c	Present results of all investigations of possible causes of heterogeneity among study results.	10-13
	20d	Present results of all sensitivity analyses conducted to assess the robustness of the synthesized results.	NA
Reporting biases	21	Present assessments of risk of bias due to missing results (arising from reporting biases) for each synthesis assessed.	NA
Certainty of evidence	22	Present assessments of certainty (or confidence) in the body of evidence for each outcome assessed.	NA
**DISCUSSION**			
Discussion	23a	Provide a general interpretation of the results in the context of other evidence.	13-17
	23b	Discuss any limitations of the evidence included in the review.	17
	23c	Discuss any limitations of the review processes used.	17
	23d	Discuss implications of the results for practice, policy, and future research.	17-18
**OTHER INFORMATION**			
Registration and protocol	24a	Provide registration information for the review, including register name and registration number, or state that the review was not registered.	NA
	24b	Indicate where the review protocol can be accessed, or state that a protocol was not prepared.	7
	24c	Describe and explain any amendments to information provided at registration or in the protocol.	NA
Support	25	Describe sources of financial or non-financial support for the review, and the role of the funders or sponsors in the review.	1
Competing interests	26	Declare any competing interests of review authors.	Other document
Availability of data, code and other materials	27	Report which of the following are publicly available and where they can be found: template data collection forms; data extracted from included studies; data used for all analyses; analytic code; any other materials used in the review.	Other document

From: Page MJ, McKenzie JE, Bossuyt PM, Boutron I, Hoffmann TC, Mulrow CD, et al. The PRISMA 2020 statement: an updated guideline for reporting systematic reviews. BMJ 2021;372:n71. doi: 10.1136/bmj.n71

For more information, visit: http://www.prisma-statement.org/

**Supplementary Table 2: Characteristics of the included studies**									
First author, date	n control	n patients	Age control	Age patients	Sex ratio control	Sex ratio patients	Short/long term abstinence	Task name	Cognitive process
Fukushima 2020	15	24	46,7	47,5	67%	71%	Long	Cue Reactivity Task	Craving
Aloi 2018	33	49	15,6	16,4	64%	69%	Long	Affective Stroop task	Emotion
Blair 2021	NR	102	NR	16,5	NR	60%	Long	The Retaliation Task	Other
Hu 2015	70	24	35,1	38,7	61%	75%	Long	Stop signal	Executive
Aloi 2021	NR	112	NR	16,3	NR	52%	Long	Comparative Optimism task	Emotion
Aloi 2020	NR	154	NR	16,1	NR	58%	Long	Passive Avoidance Task	Reward
Aloi 2021-1	NR	151	NR	16,7	NR	31%	Long	Passive Avoidance Task	Reward
Bagga 2014	18	18	35,2	36,5	100%	100%	Long	Abstract reasoning task	Executive
van Holst 2014	19	19	40,4	42,5	100%	100%	Long	Card guessing task	Decision Making
Desmond 2003	13	10	55,6	50	100%	100%	Long	Verbal Working Memory task	Executive
Pfefferbaum 2001	7	10	60,2	58,1	100%	100%	Long	N-back	Executive
Pitel 2012	12	12	43	38	100%	100%	Long	Associative Recognition task	Other
Schulte 2012	17	18	50	51	100%	100%	Long	Stroop Match-to-Sample Task	Executive
Chanraud 2011	15	15	47,7	40,1	NR	NR	Long	Working Memory Task	Executive
Forbes 2014	24	24	27,2	27,2	58%	63%	Long	card-guessing game	Reward
de Greck 2008	10	10	34,3	38,7	80%	80%	Long	Gambling-Food-	Reward

								Alcohol cue Paradigm	
Akine 2007	9	9	32,6	34,6	78%	88%	Long	False recognition task	Other
Kose 2015	13	13	33,19	37,77	54%	77%	Long	Aggression Testing (PSAP)	Other
Chanraud 2009	12	12	45	49	100%	100%	Long	Auditory language task	Other
Müller-Oehring 2019	18	21	49,6	50,3	56%	76%	Long	Addiction-Stroop color match-to-sample task	Craving
Romanczuk-Seiferth 2014	21	15	37,41	45,4	100%	100%	Long	Monetary Incentive Delay Task	Reward
Lee 2013	25	17	34	34,7	71%	72%	Long	Craving paradigm	Craving
Wiers 2015	21	22	41,95	42,14	100%	100%	Long	Stop-signal task	Executive
Grüsser 2004	10	10	41	41	50%	50%	Long	Visual alcohol-associated and control cues	Craving
Seo 2013	30	45	34,47	37,73	70%	78%	Long	Alcohol-stress cue task	Craving
Musial 2023	76	75	43,62	50,03	72%	40%	Long	Monetary Incentive Delay Task	Other
Arcurio 2013	16	15	20,25	21,2	0%	0%	Short	Decisions-to-drink	Decision Making
Claus 2011	NR	160	NR	NR	NR	71%	Short	Delay discounting task	Reward
Wesley 2017	11	23	28,8	33,3	36%	70%	Short	N-back	Executive
Heinz 2007	12	12	40	39	50%	50%	Short	Affective and alcohol associated pictures	Craving, Emotions

Bagga 2013	18	16	35,25	36,55	100%	100%	Short	Semantic judgment task	Other
Beylergil 2017	26	34	41,92	44,73	100%	100%	Short	Reward-guided decision-making task/ Affective and alcohol associated pictures	Decision Making
Burnette 2021	16	16	30,94	31	69%	69%	Short	Balloon Analog Risk Task	Decision Making
Zehra 2019	23	19	46,7	47,1	56%	74%	Short	Visual attention task	Executive
Yoon 2009	12	12	31	32	67%	75%	Short	Words and faces encoding task	Other
Czapla 2016	21	19	41,95	51,21	81%	90%	Short	Go/No-Go	Executive
Tapert 2001	10	10	21,5	19,6	0%	0%	Short	Spatial working memory and vigilance tasks	Executive
Tapert 2004	19	15	16,50	16,77	58%	67%	Short	spatial working memory and vigilance tasks	Executive
Tapert 2003	15	15	16,35	16,96	60%	60%	Short	Alcoholic beverage pictures task	Craving
Kim 2014	26	38	43,9	41,6	77%	71%	Short	Cue reactivity tasks	Craving
Maurage 2013 (1)	14	14	43	43,5	100%	100%	Short	Emotional categorization task	Emotion
Muench 2019	43	43	38,85	45,95	44%	67%	Short	Fear conditioning and extinction paradigm	Emotion

Li 2009	24	24	35,5	38,7	75%	75%	Short	stop-signal paradigm	Executive
Claus 2012	NR	144	NR	32,64	NR	NR	Short	Go/No-Go	Executive
Gerhardt 2021	15	15	41,9	47	60%	86%	Short	Simon-task	Executive
Schad 2019	24	31	42,17	42,29	88%	87%	Short	Pavlovian-instrumental transfer task	Craving
Wrase 2006	16	16	39,94	42,4	100%	100%	Short	Monetary Incentive Delay Task / Alcohol paradigm	Craving, Reward, Other
Hermann 2006	9	9	41,8	40,2	100%	100%	Short	Block-design paradigm	Other
Beck 2009	19	19	41,68	41,84	100%	100%	Short	Monetary Incentive Delay Task	Reward
Gilman 2015	18	18	30,5	30,67	67%	66%	Short	Risk-taking task	Decision Making, Reward, Other
Maurage 2012	22	22	45,1	47,2	100%	100%	Short	Cyberball task	Other
Schneider 2001	10	10	41,1	41,4	100%	100%	Short	Olfactory Stimulation	Craving
Yang 2013	15	15	45,5	42,3	100%	100%	Short	Anticipatory anxiety paradigm	Emotion
Gilman 2010	15	15	33,3	35,2	53%	53%	Short	Affective Pictures	Emotion, Executive
Sjoerd 2014	15	30	46,8	46,5	73%	53%	Short	Cue Reactivity Paradigm	Craving
Grodin 2016	17	17	27,72	32,25	53%	64%	Short	Effort cue presentation	Reward
May 2020	18	13	16,33	16,62	NR	NR	Short	Alcohol cue reactivity paradigm	Craving
Park 2011	10	11	32,7	23,9	100%	100%	Short	N-back	Executive
Schweinsburg 2005	19	15	16,5	16,91	58%	67%	Short	Spatial working memory task	Executive
Hornoiu 2023	36	49	41,8	45,5	100%	100%	Short	Neural alcohol cue	Craving

								reactivity task	
Cousijn 2023	NR	101	NR	NR	100%	100%	Short	Olfactory alcohol cue-reactivity task	Craving
Kwon 2024	28	21	22,6	23,73	NR	NR	Short	Two-stage task	Other
Le 2024	41	41	35,7	38,8	59%	80%	Short	Probabilistic learning go/no-go task	Executive
Reiter 2016	35	43	42	44,42	71%	79%	Mixed	Neural signatures of inference	Executive
Jansen 2019	39	39	44,05	41,64	56%	66%	Mixed	Emotion reappraisal task	Emotions
Stein 2021	14	13	37,71	45,62	60%	77%	Mixed	Go/no-go	Executive
Tan 2023	229	238	45,7	47	78%	78%	Mixed	Alcohol cue reactivity task	Craving

REFERENCES

1. Fukushima, S.; Kuga, H.; Oribe, N.; Mutou, T.; Yuzuriha, T.; Ozawa, H.; Ueno, T. Behavioural Cue Reactivity to Alcohol-Related and Non-Alcohol-Related Stimuli among Individuals with Alcohol Use Disorder: An FMRI Study with a Visual Task. *PLOS ONE* **2020**, *15*, e0229187, doi:https://doi.org/10.1371/journal.pone.0229187.
2. Aloi, J.; Blair, K.S.; Crum, K.I.; Meffert, H.; White, S.F.; Tyler, P.M.; Thornton, L.C.; Mobley, A.M.; Killanin, A.D.; Adams, K.O.; et al. Adolescents Show Differential Dysfunctions Related to Alcohol and Cannabis Use Disorder Severity in Emotion and Executive Attention Neuro-Circuitries. *NeuroImage: Clinical* **2018**, *19*, 782–792, doi:https://doi.org/10.1016/j.nicl.2018.06.005.
3. Blair, R.J.R.; Bajaj, S.; Sherer, N.; Bashford-Largo, J.; Zhang, R.; Aloi, J.; Hammond, C.; Lukoff, J.; Schwartz, A.; Elowsky, J.; et al. Alcohol Use Disorder and Cannabis Use Disorder Symptomatology in Adolescents and Aggression: Associations with Recruitment of Neural Regions Implicated in Retaliation. *Biological Psychiatry: Cognitive Neuroscience and Neuroimaging* **2021**, *6*, 536–544, doi:https://doi.org/10.1016/j.bpsc.2020.11.016.
4. Hu, S.; Ide, J.S.; Zhang, S.; Sinha, R.; Li, C.R. Conflict Anticipation in Alcohol Dependence — a Model-Based FMRI Study of Stop Signal Task. *NeuroImage: Clinical* **2015**, *8*, 39–50, doi:https://doi.org/10.1016/j.nicl.2015.03.008.

5. Aloi, J.; Crum, K.I.; Blair, K.S.; Zhang, R.; Johannah Bashford-Largo; Bajaj, S.; Schwartz, A.; Carollo, E.; Hwang, S.; Leiker, E.; et al. Individual Associations of Adolescent Alcohol Use Disorder versus Cannabis Use Disorder Symptoms in Neural Prediction Error Signaling and the Response to Novelty. *Developmental Cognitive Neuroscience* **2021**, *48*, 100944–100944, doi:https://doi.org/10.1016/j.dcn.2021.100944.
6. Aloi, J.; Blair, K.; Crum, K.I.; Johannah Bashford-Largo; Zhang, R.; Lukoff, J.; Carollo, E.; White, S.F.; Hwang, S.; Filbey, F.M.; et al. Alcohol Use Disorder, but Not Cannabis Use Disorder, Symptomatology in Adolescents Is Associated with Reduced Differential Responsiveness to Reward versus Punishment Feedback during Instrumental Learning. *Biological Psychiatry: Cognitive Neuroscience and Neuroimaging* **2020**, *5*, 610–618, doi:https://doi.org/10.1016/j.bpsc.2020.02.003.
7. Aloi, J.; Blair, K.S.; Meffert, H.; White, S.F.; Hwang, S.; Tyler, P.M.; Crum, K.I.; Thornton, L.C.; Mobley, A.; Killanin, A.D.; et al. Alcohol Use Disorder and Cannabis Use Disorder Symptomatology in Adolescents Is Associated with Dysfunction in Neural Processing of Future Events. *Addiction Biology* **2020**, *26*, doi:https://doi.org/10.1111/adb.12885.
8. Bagga, D.; Singh, N.; Singh, S.; Modi, S.; Kumar, P.; Bhattacharya, D.; Garg, M.L.; Khushu, S. Assessment of Abstract Reasoning Abilities in Alcohol-Dependent Subjects: An FMRI Study. *Neuroradiology* **2013**, *56*, 69–77, doi:https://doi.org/10.1007/s00234-013-1281-3.

9. van Holst, R.J.; Clark, L.; Veltman, D.J.; van den Brink, W.; Goudriaan, A.E. Enhanced Striatal Responses during Expectancy Coding in Alcohol Dependence. *Drug and Alcohol Dependence* **2014**, *142*, 204–208, doi:https://doi.org/10.1016/j.drugalcdep.2014.06.019.
10. Desmond, J.E.; Chen, S.H.Annabel.; DeRosa, E.; Pryor, M.R.; Pfefferbaum, A.; Sullivan, E.V. Increased Frontocerebellar Activation in Alcoholics during Verbal Working Memory: An FMRI Study. *NeuroImage* **2003**, *19*, 1510–1520, doi:https://doi.org/10.1016/s1053-8119(03)00102-2.
11. Pfefferbaum, A.; Desmond, J.E.; Galloway, C.; Menon, V.; Glover, G.H.; Sullivan, E.V. Reorganization of Frontal Systems Used by Alcoholics for Spatial Working Memory: An FMRI Study. *NeuroImage* **2001**, *14*, 7–20, doi:https://doi.org/10.1006/nimg.2001.0785.
12. Pitel, A.-L.; Chanraud, S.; Müller-Oehring, E.M.; Pfefferbaum, A.; Sullivan, E.V. Modulation of Limbic-Cerebellar Functional Connectivity Enables Alcoholics to Recognize Who Is Who. *Brain Structure and Function* **2012**, *218*, 683–695, doi:https://doi.org/10.1007/s00429-012-0421-6.
13. Schulte, T.; Müller-Oehring, E.M.; Sullivan, E.V.; Pfefferbaum, A. Synchrony of Corticostriatal-Midbrain Activation Enables Normal Inhibitory Control and Conflict Processing in Recovering Alcoholic Men. *Biological Psychiatry* **2012**, *71*, 269–278, doi:https://doi.org/10.1016/j.biopsych.2011.10.022.
14. Chanraud, S.; Pitel, A.-L. .; Pfefferbaum, A.; Sullivan, E.V. Disruption of Functional Connectivity of the Default-Mode Network in

Alcoholism. *Cerebral Cortex* **2011**, *21*, 2272–2281, doi:https://doi.org/10.1093/cercor/bhq297.

15. Forbes, E.E.; Rodriguez, E.E.; Musselman, S.; Narendran, R. Prefrontal Response and Frontostriatal Functional Connectivity to Monetary Reward in Abstinent Alcohol-Dependent Young Adults. *PLoS ONE* **2014**, *9*, e94640, doi:https://doi.org/10.1371/journal.pone.0094640.
16. de Greck, M.; Supady, A.; Thiemann, R.; Tempelmann, C.; Bogerts, B.; Forschner, L.; Ploetz, K. v.; Northoff, G. Decreased Neural Activity in Reward Circuitry during Personal Reference in Abstinent Alcoholics-A FMRI Study. *Human Brain Mapping* **2009**, *30*, 1691–1704, doi:https://doi.org/10.1002/hbm.20634.
17. Akine, Y.; Kato, M.; Muramatsu, T.; Umeda, S.; Mimura, M.; Asai, Y.; Tanada, S.; Obata, T.; Hiroo Ikehira; Kashima, H.; et al. Altered Brain Activation by a False Recognition Task in Young Abstinent Patients with Alcohol Dependence. *Alcoholism Clinical and Experimental Research* **2007**, *31*, 1589–1597, doi:https://doi.org/10.1111/j.1530-0277.2007.00453.x.
18. Kose, S.; Steinberg, J.L.; Moeller, F.G.; Gowin, J.L.; Zuniga, E.; Kamdar, Z.N.; Schmitz, J.M.; Lane, S.D. Neural Correlates of Impulsive Aggressive Behavior in Subjects with a History of Alcohol Dependence. *Behavioral Neuroscience* **2015**, *129*, 183–196, doi:https://doi.org/10.1037/bne0000038.
19. Chanraud-Guillermo, S.; Andoh, J.; Martelli, C.; Artiges, E.; Christophe Pallier; Aubin, H.; Jean-Luc Martinot; Reynaud, M. Imaging of Language-

Related Brain Regions in Detoxified Alcoholics. *Alcoholism Clinical and Experimental Research* **2009**, *33*, 977–984, doi:https://doi.org/10.1111/j.1530-0277.2009.00918.x.

20. Müller-Oehring, E.M.; Le Berre, A.-P.; Serventi, M.; Kalon, E.; Haas, A.L.; Padula, C.B.; Schulte, T. Brain Activation to Cannabis- and Alcohol-Related Words in Alcohol Use Disorder. *Psychiatry Research: Neuroimaging* **2019**, *294*, 111005, doi:https://doi.org/10.1016/j.pscychresns.2019.111005.
21. Romanczuk-Seiferth, N.; Koehler, S.; Dreesen, C.; Wüstenberg, T.; Heinz, A. Pathological Gambling and Alcohol Dependence: Neural Disturbances in Reward and Loss Avoidance Processing. *Addiction Biology* **2014**, *20*, 557–569, doi:https://doi.org/10.1111/adb.12144.
22. Lee, E.; Ku, J.; Jung, Y.-C.; Lee, H.; An, S.K.; Kim, K.R.; Yoon, K.-J.; Namkoong, K. Neural Evidence for Emotional Involvement in Pathological Alcohol Craving. *Alcohol and Alcoholism* **2013**, *48*, 288–294.
23. Wiers, C.E.; Gawron, C.K.; Gröpper, S.; Spengler, S.; Stuke, H.; Lindenmeyer, J.; Walter, H.; Bermpohl, F. Decreased Gray Matter Volume in Inferior Frontal Gyrus Is Related to Stop-Signal Task Performance in Alcohol-Dependent Patients. *Psychiatry Research: Neuroimaging* **2015**, *233*, 125–130, doi:https://doi.org/10.1016/j.pscychresns.2015.05.006.
24. Grüsser, S.M.; Wrase, J.; Klein, S.; Hermann, D.; Smolka, M.N.; Ruf, M.; Weber-Fahr, W.; Flor, H.; Mann, K.; Braus, D.F.; et al. Cue-Induced Activation of the Striatum and Medial Prefrontal Cortex Is Associated with

Subsequent Relapse in Abstinent Alcoholics. *Psychopharmacology* **2004**, *175*, 296–302, doi:https://doi.org/10.1007/s00213-004-1828-4.

25. Seo, D.; Lacadie, C.M.; Tuit, K.; Hong, K.-I.; Constable, R.T.; Sinha, R. Disrupted Ventromedial Prefrontal Function, Alcohol Craving, and Subsequent Relapse Risk. *JAMA Psychiatry* **2013**, *70*, 727, doi:https://doi.org/10.1001/jamapsychiatry.2013.762.
26. Musial, M.P.M.; Beck, A.; Rosenthal, A.; Charlet, K.; Bach, P.; Kiefer, F.; Vollstädt-Klein, S.; Walter, H.; Heinz, A.; Rothkirch, M. Reward Processing in Alcohol-Dependent Patients and First-Degree Relatives: Functional Brain Activity during Anticipation of Monetary Gains and Losses. *Biological Psychiatry* **2023**, *93*, 546–557, doi:https://doi.org/10.1016/j.biopsych.2022.05.024.
27. Arcurio, L.R.; Finn, P.R.; James, T.W. Neural Mechanisms of High-Risk Decisions-To-Drink in Alcohol-Dependent Women. *Addiction Biology* **2013**, *20*, 390–406, doi:https://doi.org/10.1111/adb.12121.
28. Claus, E.D.; Kiehl, K.A.; Hutchison, K.E. Neural and Behavioral Mechanisms of Impulsive Choice in Alcohol Use Disorder. *Alcoholism: Clinical and Experimental Research* **2011**, *35*, 1209–1219, doi:https://doi.org/10.1111/j.1530-0277.2011.01455.x.
29. Wesley, M.J.; Lile, J.A.; Fillmore, M.T.; Porrino, L.J. Neurophysiological Capacity in a Working Memory Task Differentiates Dependent from Nondependent Heavy Drinkers and Controls. *Drug and Alcohol*

Dependence **2017**, *175*, 24–35, doi:https://doi.org/10.1016/j.drugalcdep.2017.01.029.
30. Heinz, A.; Wrase, J.; Kahnt, T.; Beck, A.; Bromand, Z.; Grüsser, S.M.; Kienast, T.; Smolka, M.N.; Flor, H.; Mann, K. Brain Activation Elicited by Affectively Positive Stimuli Is Associated with a Lower Risk of Relapse in Detoxified Alcoholic Subjects. *Alcoholism: Clinical and Experimental Research* **2007**, *31*, 1138–1147, doi:https://doi.org/10.1111/j.1530-0277.2007.00406.x.
31. Bagga, D.; Singh, N.; Modi, S.; Kumar, P.; Bhattacharya, D.; Garg, M.; Khushu, S. Assessment of Lexical Semantic Judgment Abilities in Alcohol-Dependent Subjects: An FMRI Study. *Journal of Biosciences* **2013**, *38*, 905–915, doi:https://doi.org/10.1007/s12038-013-9387-7.
32. Beylergil, S.B.; Beck, A.M.; Heinz, A.; Lorenz, R.D.; Rapp, M.A.; Schlagenhauf, F.; Heinz, A.; Obermayer, K. Dorsolateral Prefrontal Cortex Contributes to the Impaired Behavioral Adaptation in Alcohol Dependence. *NeuroImage: Clinical* **2017**, *15*, 80–94, doi:https://doi.org/10.1016/j.nicl.2017.04.010.
33. Burnette, E.M.; Grodin, E.N.; Ghahremani, D.G.; Galván, A.; Kohno, M.; Ray, L.A.; London, E.D. Diminished Cortical Response to Risk and Loss during Risky Decision Making in Alcohol Use Disorder. *Drug and Alcohol Dependence* **2020**, *218*, 108391–108391, doi:https://doi.org/10.1016/j.drugalcdep.2020.108391.

34. Zehra, A.; Lindgren, E.; Wiers, C.E.; Freeman, C.; Miller, G.; Ramirez, V.; Shokri-Kojori, E.; Wang, G.-J.; Talagala, L.; Tomasi, D.; et al. Neural Correlates of Visual Attention in Alcohol Use Disorder. *Drug and Alcohol Dependence* **2019**, *194*, 430–437, doi:https://doi.org/10.1016/j.drugalcdep.2018.10.032.
35. Yoon, H.W.; Chung, J.-Y.; Oh, J.-H.; Min, H.-K.; Kim, D.-J.; Cheon, Y.; Joe, K.H.; Kim, Y.-B.; Cho, Z.-H. Differential Activation of Face Memory Encoding Tasks in Alcohol-Dependent Patients Compared to Healthy Subjects: An FMRI Study. *Neuroscience Letters* **2009**, *450*, 311–316, doi:https://doi.org/10.1016/j.neulet.2008.12.011.
36. Czapla, M.; Baeuchl, C.; Simon, J.J.; Richter, B.; Kluge, M.; Friederich, H.-C.; Mann, K.; Herpertz, S.C.; Loeber, S. Do Alcohol-Dependent Patients Show Different Neural Activation during Response Inhibition than Healthy Controls in an Alcohol-Related FMRI Go/No-Go-Task? *Psychopharmacology* **2017**, *234*, 1001–1015, doi:https://doi.org/10.1007/s00213-017-4541-9.
37. Tapert, S.F.; Brown, G.G.; Kindermann, S.S.; Cheung, E.H.; Frank, L.R.; Brown, S.A. FMRI Measurement of Brain Dysfunction in Alcohol-Dependent Young Women. *Alcoholism: Clinical and Experimental Research* **2001**, *25*, 236–245, doi:https://doi.org/10.1111/j.1530-0277.2001.tb02204.x.
38. Tapert, S.F.; Schweinsburg, A.D.; Barlett, V.C.; Brown, S.A.; Frank, L.R.; Brown, G.G.; Meloy, M.J. Blood Oxygen Level Dependent Response and Spatial Working Memory in Adolescents with Alcohol Use

Disorders. *Alcoholism: Clinical & Experimental Research* **2004**, *28*, 1577–1586, doi:https://doi.org/10.1097/01.alc.0000141812.81234.a6.

39. Tapert, S.F.; Cheung, E.H.; Brown, G.G.; Frank, L.R.; Paulus, M.P.; Schweinsburg, A.D.; Meloy, M.J.; Brown, S.A. Neural Response to Alcohol Stimuli in Adolescents with Alcohol Use Disorder. *Archives of General Psychiatry* **2003**, *60*, 727, doi:https://doi.org/10.1001/archpsyc.60.7.727.
40. Kim, S.M.; Han, D.H.; Min, K.J.; Kim, B.-N.; Cheong, J.H. Brain Activation in Response to Craving- and Aversion-Inducing Cues Related to Alcohol in Patients with Alcohol Dependence. *Drug and Alcohol Dependence* **2014**, *141*, 124–131, doi:https://doi.org/10.1016/j.drugalcdep.2014.05.017.
41. Maurage, P.; Joassin, F.; Pesenti, M.; Grandin, C.; Heeren, A.; Philippot, P.; de Timary, P. The Neural Network Sustaining Crossmodal Integration Is Impaired in Alcohol-Dependence: An FMRI Study. *Cortex* **2013**, *49*, 1610–1626, doi:https://doi.org/10.1016/j.cortex.2012.04.012.
42. Muench, C.; Charlet, K.; Balderston, N.L.; Grillon, C.; Heilig, M.; Cortes, C.R.; Momenan, R.; Lohoff, F.W. Fear Conditioning and Extinction in Alcohol Dependence: Evidence for Abnormal Amygdala Reactivity. *Addiction Biology***2019**, *26*, doi:https://doi.org/10.1111/adb.12835.
43. Li, C.R.; Luo, X.; Yan, P.; Bergquist, K.; Sinha, R. Altered Impulse Control in Alcohol Dependence: Neural Measures of Stop Signal Performance. *Alcoholism: Clinical and Experimental Research* **2009**, *33*, 740–750, doi:https://doi.org/10.1111/j.1530-0277.2008.00891.x.

44. Claus, E.D.; Feldstein Ewing, S.W.; Filbey, F.M.; Hutchison, K.E. Behavioral Control in Alcohol Use Disorders: Relationships with Severity. *Journal of Studies on Alcohol and Drugs* **2013**, *74*, 141–151, doi:https://doi.org/10.15288/jsad.2013.74.141.
45. Gerhardt, S.; Luderer, M.; Bumb, J.M.; Sobanski, E.; Moggi, F.; Kiefer, F.; Vollstädt-Klein, S. Stop What You're Doing!—an FMRI Study on Comparisons of Neural Subprocesses of Response Inhibition in ADHD and Alcohol Use Disorder. *Frontiers in Psychiatry* **2021**, *12*, doi:https://doi.org/10.3389/fpsyt.2021.691930.
46. Schad, D.J.; Garbusow, M.; Friedel, E.; Sommer, C.; Sebold, M.; Hägele, C.; Bernhardt, N.; Nebe, S.; Kuitunen-Paul, S.; Liu, S.; et al. Neural Correlates of Instrumental Responding in the Context of Alcohol-Related Cues Index Disorder Severity and Relapse Risk. *European Archives of Psychiatry and Clinical Neuroscience* **2018**, *269*, 295–308, doi:https://doi.org/10.1007/s00406-017-0860-4.
47. Wrase, J.; Schlagenhauf, F.; Kienast, T.; Wüstenberg, T.; Bermpohl, F.; Kahnt, T.; Beck, A.; Ströhle, A.; Juckel, G.; Knutson, B.; et al. Dysfunction of Reward Processing Correlates with Alcohol Craving in Detoxified Alcoholics. *NeuroImage* **2007**, *35*, 787–794, doi:https://doi.org/10.1016/j.neuroimage.2006.11.043.
48. Hermann, D.; Smolka, M.N.; Klein, S.; Heinz, A.; Mann, K.; Braus, D.F. IMAGING STUDY: Reduced FMRI Activation of an Occipital Area in Recently Detoxified Alcohol-Dependent Patients in a Visual and Acoustic

Stimulation Paradigm. *Addiction Biology* **2006**, *12*, 117–121, doi:https://doi.org/10.1111/j.1369-1600.2006.00039.x.

49. Beck, A.; Schlagenhauf, F.; Wüstenberg, T.; Hein, J.; Kienast, T.; Kahnt, T.; Schmack, K.; Hägele, C.; Knutson, B.; Heinz, A.; et al. Ventral Striatal Activation during Reward Anticipation Correlates with Impulsivity in Alcoholics. *NeuroImage* **2009**, *47*, S159, doi:https://doi.org/10.1016/s1053-8119(09)71675-1.
50. Gilman, J.M.; Smith, A.R.; Bjork, J.M.; Ramchandani, V.A.; Momenan, R.; Hommer, D.W. Cumulative Gains Enhance Striatal Response to Reward Opportunities in Alcohol-Dependent Patients. *Addiction Biology* **2014**, *20*, 580–593, doi:https://doi.org/10.1111/adb.12147.
51. Maurage, P.; Joassin, F.; Philippot, P.; Heeren, A.; Vermeulen, N.; Mahau, P.; Delperdange, C.; Corneille, O.; Luminet, O.; de Timary, P. Disrupted Regulation of Social Exclusion in Alcohol-Dependence: An FMRI Study. *Neuropsychopharmacology* **2012**, *37*, 2067–2075, doi:https://doi.org/10.1038/npp.2012.54.
52. Schneider, F.; Habel, U.; Wagner, M.; Franke, P.; Salloum, J.B.; Shah, N.J.; Toni, I.; Sulzbach, C.; Hönig, K.; Maier, W.; et al. Subcortical Correlates of Craving in Recently Abstinent Alcoholic Patients. *American Journal of Psychiatry* **2001**, *158*, 1075–1083, doi:https://doi.org/10.1176/appi.ajp.158.7.1075.
53. Yang, H.; Devous, M.D.; Briggs, R.W.; Spence, J.S.; Xiao, H.; Kreyling, N.; Adinoff, B. Altered Neural Processing of Threat in Alcohol-Dependent

Men. *Alcohol: Clinical & Experimental Research* **2013**, *37*, 2029–2038, doi:https://doi.org/10.1111/acer.12187.

54. Gilman, J.M.; Davis, M.B.; Hommer, D.W. Greater Activation in Left Hemisphere Language-Related Regions during Simple Judgment Tasks among Substance-Dependent Patients in Treatment for Alcoholism. *Alcoholism: Clinical and Experimental Research* **2010**, *34*, 331–341, doi:https://doi.org/10.1111/j.1530-0277.2009.01095.x.
55. Sjoerds, Z.; van den Brink, W.; Beekman, A.T.F.; Penninx, B.W.J.H.; Veltman, D.J. Cue Reactivity Is Associated with Duration and Severity of Alcohol Dependence: An FMRI Study. *PLoS ONE* **2014**, *9*, e84560, doi:https://doi.org/10.1371/journal.pone.0084560.
56. Grodin, E.N.; Steckler, L.E.; Momenan, R. Altered Striatal Response during Effort-Based Valuation and Motivation in Alcohol-Dependent Individuals. *Alcohol and Alcoholism* **2016**, *51*, 638–646, doi:https://doi.org/10.1093/alcalc/agw003.
57. May, A.C.; Jacobus, J.; Stewart, J.L.; Simmons, A.N.; Paulus, M.P.; Tapert, S.F. Do Adolescents Use Substances to Relieve Uncomfortable Sensations? A Preliminary Examination of Negative Reinforcement among Adolescent Cannabis and Alcohol Users. *Brain Sciences* **2020**, *10*, 214, doi:https://doi.org/10.3390/brainsci10040214.
58. PARK, M.-S.; SOHN, S.; PARK, J.-E.; KIM, S.-H.; YU, I.K.; SOHN, J.-H. Brain Functions Associated with Verbal Working Memory Tasks among Young Males with Alcohol Use Disorders. *Scandinavian Journal of*

Psychology **2010**, *52*, 1–7, doi:https://doi.org/10.1111/j.1467-9450.2010.00848.x.

59. Schweinsburg, A.D.; Schweinsburg, B.C.; Cheung, E.H.; Brown, G.G.; Brown, S.A.; Tapert, S.F. FMRI Response to Spatial Working Memory in Adolescents with Comorbid Marijuana and Alcohol Use Disorders. *Drug and Alcohol Dependence* **2005**, *79*, 201–210, doi:https://doi.org/10.1016/j.drugalcdep.2005.01.009.
60. Hornoiu, I.L.; Lee, A.M.; Tan, H.; Helmut Nakovics; Bach, P.; Mann, K.; Kiefer, F.; Sommer, W.H.; Vollstädt-Klein, S. The Role of Unawareness, Volition, and Neural Hyperconnectivity in Alcohol Use Disorder: A Functional Magnetic Resonance Imaging Study. *Biological Psychiatry Cognitive Neuroscience and Neuroimaging* **2022**, *8*, 660–671, doi:https://doi.org/10.1016/j.bpsc.2022.12.008.
61. Cousijn, J.; Mies, G.W.; Runia, N.; Derksen, M.; Ingo Willuhn; Heidi The Impact of Age on Olfactory Alcohol Cue-Reactivity: A Functional Magnetic Resonance Imaging Study in Adolescent and Adult Male Drinkers. *Alcoholism: Clinical and Experimental Research* **2023**, *47*, 668–677, doi:https://doi.org/10.1111/acer.15037.
62. Kwon, M.; Choi, H.; Park, H.; Ahn, W.-Y.; Jung, Y.-C. Neural Correlates of Model-Based Behavior in Internet Gaming Disorder and Alcohol Use Disorder. *Journal of Behavioral Addictions* **2024**, *13*, 236–249, doi:https://doi.org/10.1556/2006.2024.00006.

63. Le, T.; Oba, T.; Couch, L.; McInerney, L.; Li, C.-S. Deficits in Proactive Avoidance and Neural Responses to Drinking Motives in Problem Drinkers. *Research Square (Research Square)* **2024**, doi:https://doi.org/10.21203/rs.3.rs-3924584/v1.
64. Reiter, A.M.F.; Deserno, L.; Kallert, T.; Heinze, H.-J. .; Heinz, A.; Schlagenhauf, F. Behavioral and Neural Signatures of Reduced Updating of Alternative Options in Alcohol-Dependent Patients during Flexible Decision-Making. *Journal of Neuroscience* **2016**, *36*, 10935–10948, doi:https://doi.org/10.1523/jneurosci.4322-15.2016.
65. Jansen, J.M.; van den Heuvel , O.; van der Werf , Y.; Stella, S.; Veltman, D.J.; van den Brink , W.; Goudriaan, A.E. Emotion Processing, Reappraisal, and Craving in Alcohol Dependence: A Functional Magnetic Resonance Imaging Study. *Frontiers in Psychiatry* **2019**, *10*, doi:https://doi.org/10.3389/fpsyt.2019.00227.
66. Stein, M.; Steiner, L.; Fey, W.; Conring, F.; Rieger, K.; Federspiel, A.; Moggi, F. Alcohol-Related Context Modulates Neural Correlates of Inhibitory Control in Alcohol Dependent Patients: Preliminary Data from an FMRI Study Using an Alcohol-Related Go/NoGo-Task. *Behavioural Brain Research* **2021**, *398*, 112973, doi:https://doi.org/10.1016/j.bbr.2020.112973.
67. Tan, H.; Martin Fungisai Gerchen; Bach, P.; Lee, A.M.; Hummel, O.; Sommer, W.; Kirsch, P.; Kiefer, F.; Vollstädt-Klein, S. Decoding FMRI Alcohol Cue Reactivity and Its Association with Drinking Behaviour. *BMJ*

Mental Health **2023**, *26*, e300639–e300639,

doi:https://doi.org/10.1136/bmjment-2022-300639.
